# Increased Risk of NAFLD in Adults with Glomerular Hyperfiltration: An 8-Year Cohort Study Based on 147,162 Koreans

**DOI:** 10.3390/jpm12071142

**Published:** 2022-07-14

**Authors:** Dae-Jeong Koo, Mi Yeon Lee, Inha Jung, Sun Joon Moon, Hyemi Kwon, Eun-Jung Rhee, Cheol-Young Park, Won-Young Lee, Ki Won Oh, Se Eun Park

**Affiliations:** 1 Division of Endocrinology and Metabolism, Department of Internal Medicine, Changwon Fatima Hospital, 45, Changi-daero, Uichang-gu, Changwon-si 51394, Korea; newkokdj@gmail.com; 2 Division of Biostatistics, Department of R&D Management, Kangbuk Samsung Hospital, Sungkyunkwan University School of Medicine, 29, Saemunan-ro, Jongno-gu, Seoul 03181, Korea; my7713.lee@samsung.com; 3 Division of Endocrinology and Metabolism, Department of Internal Medicine, Korea University Ansan Hospital, College of Medicine, Korea University, 123, Jeokgeum-ro, Danwon-gu, Ansan-si 15355, Korea; inhajung129@gmail.com; 4 Division of Endocrinology and Metabolism, Department of Internal Medicine, Kangbuk Samsung Hospital, Sungkyunkwan University School of Medicine, 29, Saemunan-ro, Jongno-gu, Seoul 03181, Korea; ipleat.m@gmail.com (S.J.M.); hyemi.kwon@samsung.com (H.K.); hongsiri@hanmail.net (E.-J.R.); cydoctor@chol.com (C.-Y.P.); drlwy@hanmail.net (W.-Y.L.); okwendo@naver.com (K.W.O.)

**Keywords:** nonalcoholic fatty liver disease, liver fibrosis, glomerular filtration rate, obesity, insulin resistance, cohort study

## Abstract

This study evaluated whether glomerular hyperfiltration (GHF) could predict nonalcoholic fatty liver disease (NAFLD) and fibrosis. A longitudinal cohort study including 147,479 participants aged 20–65 years without NAFLD and kidney disease at baseline was performed. GHF cutoff values were defined as age- and sex-specific estimated glomerular filtration rate (eGFRs) above the 95th percentile, and eGFR values between the 50th and 65th percentiles were used as reference groups. NAFLD was diagnosed via abdominal ultrasonography, and the fibrosis status was evaluated using the NAFLD fibrosis score and Fibrosis-4. During 598,745 person years of follow-up (median, 4.6 years), subjects with GHF at baseline had the highest hazard ratio (HR) for the development of NAFLD (HR 1.21; 95% CI 1.14–1.29) and fibrosis progression (HR 1.42; 95% CI 1.11–1.82) after adjusting for confounding factors. A higher baseline eGFR percentile maintained a higher risk of NAFLD and fibrosis probability. The persistent GHF group during follow-up had the highest HR for NAFLD compared to the persistent non-GHF group (HR 1.31; 95% CI 1.14–1.51). These results were consistent in all subgroups and statistically more prominent in participants without diabetes. GHF was positively associated with increased risk of NAFLD and probability of liver fibrosis in healthy adults.

## 1. Introduction

Nonalcoholic fatty liver disease (NAFLD) has already become the most common chronic liver disease diagnosed by imaging, with a worldwide prevalence of 25% [1,2]. NAFLD is a potent clinical feature for insulin resistance (IR) and metabolic syndrome (MetS) and is considered a proven risk factor for type 2 diabetes [3]. NAFLD ranges from simple steatosis to progressive nonalcoholic steatohepatitis, which is associated with high mortality due to complications from liver disorders and cardiovascular disease [4,5]. From this point of view, finding an easy way to diagnose or predict the development of NAFLD at an early stage has long been a clinically important research endpoint.

Kidney function and hepatic conditions are mutually connected. Acute or chronic liver disease causes acute or chronic kidney dysfunction and vice versa [6,7]. The most common examples of interconnected organ dysfunction are NAFLD with chronic kidney disease (CKD) and acute kidney injury with liver dysfunction [6]. Previous studies have suggested that IR, inflammatory cytokine production, activation of the renin–angiotensin system, redox imbalance, and the impact of these on the epigenetic regulation of gene expression might play roles [8,9,10].

Glomerular hyperfiltration (GHF), an abnormally high glomerular filtration rate (GFR), is a phenomenon that can occur during various clinical conditions including kidney disease. Increased GFR can occur as an early manifestation of diseases such as diabetes mellitus, but whether GHF is a precursor of CKD remains to be proven [11]. Recently, the complex relationship between GHF and metabolic disease has been a focus of clinical attention. GHF is associated with various clinical outcomes, including obesity [12], prediabetes [13], cardiovascular disease [14], dementia [15], and other MetS [16]. GHF was reported to be associated with liver disease severity in 179 children with histologically confirmed NAFLD [17], and GHF was also associated with body weight and presence of NAFLD in adults with MetS [18]. Although GHF is associated with several metabolic disorders, few longitudinal studies based on large cohorts have focused on the association between GHF and fatty liver and its fibrosis progression.

Thus, we performed a longitudinal study to evaluate the relative diagnostic value of baseline GHF for the development of NAFLD and fibrosis probability in a large Korean occupational cohort. We also evaluated whether changes in the hyperfiltration status might be associated with the development of NAFLD during the follow-up period.

## 2. Materials and Methods

### 2.1. Study Population and Design

**Figure 1:**
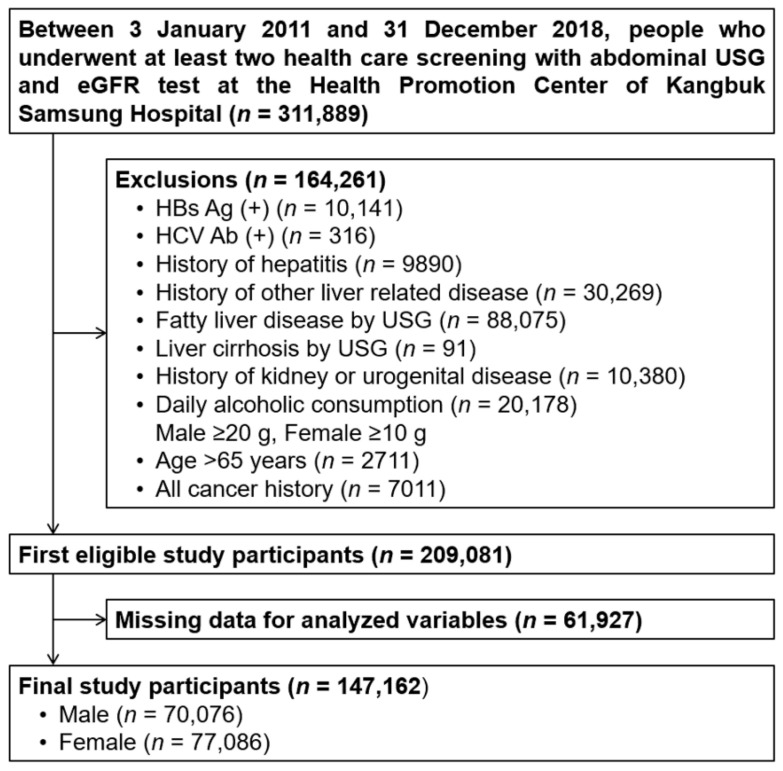
Diagram of study enrollment. eGFR: estimated glomerular filtration rate, HBs Ag: hepatitis B virus surface antigen, HCV Ab: hepatitis C virus antibody, USG: ultrasonography.

The Kangbuk Samsung Health Study was a cohort study of South Korean adults who underwent a comprehensive annual or biennial health examination at the Kangbuk Samsung Hospital Total Healthcare Center in Seoul and Suwon, Korea [19]. This study was conducted in adults aged 20–65 years who had regular medical examinations. Between 3 January 2011 and 31 December 2018, 311,889 subjects who underwent abdominal ultrasonography (USG) and estimated GFR (eGFR) were initially selected. Among these, subjects with any of the following conditions at baseline were excluded: liver cirrhosis (*n* = 91) and NAFLD (*n* = 88,075) confirmed by abdominal USG at the initial examination; positive serologic markers for hepatitis B (*n* = 10,141) or hepatitis C virus (*n* = 316); a history of other liver-related diseases (*n* = 30,269); an alcohol intake of ≥20 g/day for men and ≥10 g/day for women (*n* = 20,178); a history of kidney disease including CKD Stage III (eGFR < 60 mL/min/1.73 m^2^) or urogenital disease (*n* = 10,380); a history of any cancer (*n* = 7011); and an age of >65 years (*n* = 2711). Subjects with any missing data for the analyzed variables were excluded (*n* = 61,927). After exclusions, a total of 147,162 participants were included in the study (70,076 males and 77,086 females) (Figure 1). The study participants underwent a follow-up after the first visit, and at least one additional visit was made. During the follow-up, if NAFLD was diagnosed, the follow-up was terminated. Therefore, data from various timepoints in that period are included. The average follow-up period was 4.5 ± 1.9 years, mean ± standard deviation (SD). This study was approved by the Institutional Review Board of Kangbuk Samsung Hospital (KBSMC 202003310001), which waived the requirement for informed consent because only retrospectively accessed, de-identified data were analyzed.

### 2.2. Anthropometric and Laboratory Measurements

All information about the study subjects was acquired through a self-administered questionnaire. The questionnaire was prepared based on the 4th National Health and Nutrition Survey [20] and the Korean version of the International Physical Activity Questionnaire short form [21]. Regular exercise was considered as performance of moderate or high intensity exercise more than three times a week. Body weight was measured while the subjects were wearing a thin gown, and body mass index (BMI) was calculated by dividing the body weight (kg) by the height squared (m^2^). Blood pressure (BP) was measured on both arms at an interval of 1 min or more while the subjects were in a sitting position. A standard sphygmomanometer was used when the participants were in a stable state. The higher measured value was considered as BP. All biochemical blood sampling was conducted after fasting for more than 12 h. To assess IR, the homeostatic model assessment of IR (HOMA-IR) was used [22]. Additionally, high-sensitivity C-reactive protein (hs-CRP) was applied as an indicator of inflammation [23]. Dyslipidemia was defined as total cholesterol ≥00 mg/dL, HDL cholesterol levels <40 mg/dL in men and <50 mg/dL in women, triglyceride levels ≥150 mg/dL, LDL cholesterol levels ≥130 mg/dL, or use of antidyslipidemic drugs. Obesity was defined as a BMI ≥25 kg/m^2^. Hypertension (HT) was defined as systolic BP ≥140 mm Hg or diastolic BP ≥90 mm Hg, having a history of hypertension, or currently taking anti-hypertensive agents. Underlying diabetes mellitus (DM) was defined as fasting blood glucose levels ≥126 mg/dL and HbA1c ≥6.5%, having a history of diabetes, or currently taking anti-diabetic agents [24].

### 2.3. Diagnosis of NAFLD and Advanced Liver Fibrosis

Fatty liver was defined by evidence of hepatic steatosis on USG, such as higher liver echogenicity than that of the renal cortex and spleen, attenuation of ultrasound waves, loss of definition of the diaphragm, and poor delineation of the intrahepatic architecture [25]. Liver USG was performed by a well-trained examiner, and the final diagnosis was made by experienced multiple radiologists who were not aware of the purpose of this study. In our center, the kappa values for the diagnosis of fatty liver have high reliability with an inter-observer agreement of 0.74 and an intra-observer agreement of 0.94 [26]. NAFLD was defined as a fatty liver without other etiologies of hepatic steatosis as described in the exclusion criteria [1].

To evaluate the probability of advanced liver fibrosis in patients with NAFLD, the NAFLD fibrosis score (NFS) and Fibrosis-4 (FIB-4) were applied [27]. NFS and FIB-4 were calculated using the following formulas: NFS = −1.675 + 0.037 × age (years) + 0.094 × BMI (kg/m^2^) + 1.13 × impaired fasting glucose (IFG) or diabetes (yes = 1, no = 0) + 0.99 × aspartate aminotransferase (AST) (U/L)/alanine aminotransferase (ALT) (U/L) − 0.013 × platelet (10^9^/L) − 0.66 × albumin (g/dL); FIB-4 = age (years) × AST (U/L)/[platelet (10^9^/L) × ALT ^½^ (U/L)]. The cutoff value to exclude the presence of advanced liver fibrosis was −1.455 for NFS and 1.30 for FIB-4 [28].

### 2.4. Definition of Glomerular Hyperfiltration

In this study, we used the Chronic Kidney Disease Epidemiology Collaboration (CKD-EPI) creatinine equation to acquire the eGFR [29]. The GFR is affected by age, sex, lean body mass, and presence of diabetes or hypertension. Therefore, rather than defining GHF as a uniform absolute value, a relative value according to the study group was applied, in consideration of the biological and metabolic status of the study subjects. We defined the GHF cutoff values as an eGFR with residuals above the 95th percentile rather than the 90th percentile for each specific group [30]. We calculated the eGFR values corresponding to values over the 95th percentile in each of the groups to evaluate age- and sex-specific hyperfiltration. The male and female subjects were further divided into five-year age interval groups. The participants were also divided into 15 eGFR percentile interval groups from the 5th to the 94th percentile, in addition to the eGFR percentile groups corresponding to values below the 5th percentile and above the 95th percentile [15]. 

### 2.5. Statistical Analysis

Continuous data were expressed as the mean ± standard deviation and were compared using a *t*-test for two independent samples, as appropriate. In the case of skewed distributions, the median (interquartile range) was considered, and the Mann–Whitney U test was used. One-way analysis of variance (ANOVA) for more than two continuous independent variables and the Kruskal–Wallis test for non-normally distributed variables were conducted.

The primary endpoint of this study was the development of incident NAFLD diagnosed by abdominal USG. To estimate the hazard ratio (HR) with 95% confidence interval (CI) for the development of NAFLD, a Cox proportional hazard model was used according to the eGFR percentile group at baseline. The reference group had eGFR values between the 50th and the 64th percentiles in each group, which was the median distribution of the study subjects. To analyze the association between baseline eGFR and incidence of NAFLD, a parametric proportional hazards model with 95% CI, allowing the for interval-censored events, was applied [31], and restricted cubic splines were used to estimate the baseline log-cumulative hazards. Local regression smoothing in a generalized additive model was used to show curves of pointwise estimation and CI of HR of the residuals of eGFR. Compounding factors related to NAFLD and renal function were adjusted.

In addition, the study participants were divided into subgroups corresponding to the confounding factors associated with the development of NAFLD: age (20 years ≤ age < 40 years, 40 years ≤ age < 65 years), sex, BMI (<25, ≥25 kg/m^2^), exercise status (<3 times/week, ≥3 times/week, or unknown), smoking status (current, non-current, or unknown), diabetes (yes, no), hypertension (yes, no), dyslipidemia (yes, no), and HOMA-IR (<50 percentile, ≥50 percentile of study participants). Additionally, we performed predefined subgroup analyses. Confounder-adjusted Model 3 was applied in all subgroup analyses.

All reported 2-tailed *p*-values of <0.05 were considered statistically significant. All statistical analyses were performed using STATA version 16.1 (StataCorp, College Station, TX, USA).

## 3. Results

In total, 147,162 subjects consisting of 70,076 males and 77,086 females, were included in this study. During 598,745 person years of follow-up (median follow-up period 4.6 years), a total of 29,410 subjects developed NAFLD. Of the study subjects, 7369 were categorized into the hyperfiltration group. Among the eight eGFR percentile interval groups, participants with GHF at baseline had a higher IR index and included a higher proportion of subjects who did not exercise (Supplementary Materials Table S1). The median (interquartile range) eGFR value of the entire participants was 106.5 (94.0–114.5) mL/min/1.73 m^2^. The baseline characteristics according to the development of NAFLD at the end of the follow-up are shown in Table 1. Compared to subjects without NAFLD, those who developed NAFLD were older, more obese, and had higher levels of liver enzymes and poorer lipid profiles. The proportion of subjects with comorbid diabetes and hypertension was also higher in the NAFLD group. However, the mean baseline eGFR value was rather low in subjects that would develop NAFLD. This appeared to be due to the effect of the difference in mean age, sex composition, and total *n* value between the two groups. The mean age was higher in the group with NAFLD. The cut-off values based on the definition of GHF in this study showed a tendency to decrease rapidly with increasing age in both men and women (Supplementary Materials Figure S1). 

**Figure 2:**
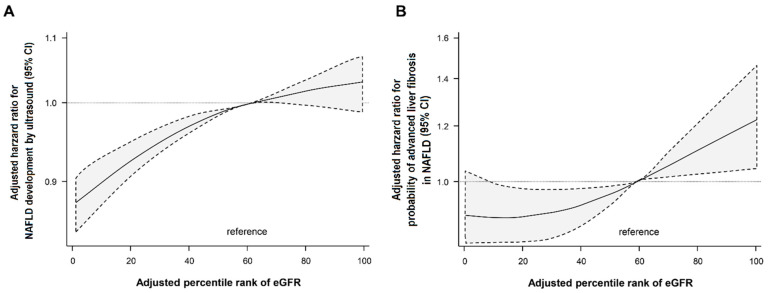
HR for the development of NAFLD (**A**) and advanced liver fibrosis of NAFLD based on NFS (**B**) according to baseline eGFR percentile. Adjusted for age, sex, muscle mass, BMI, smoking status, regular exercise, history of diabetes, dyslipidemia, hypertension, hs-CRP, and HOMA-IR. BMI: body mass index, eGFR: estimated glomerular filtration rate, GHF: glomerular hyperfiltration, HOMA-IR: homeostasis model assessment of insulin resistance, HR: hazard ratio, hs-CRP: high-sensitivity C-reactive protein, NAFLD: nonalcoholic fatty liver disease, NFS: NAFLD fibrosis score, Non-GHF: non-glomerular hyperfiltration.

**Figure 3:**
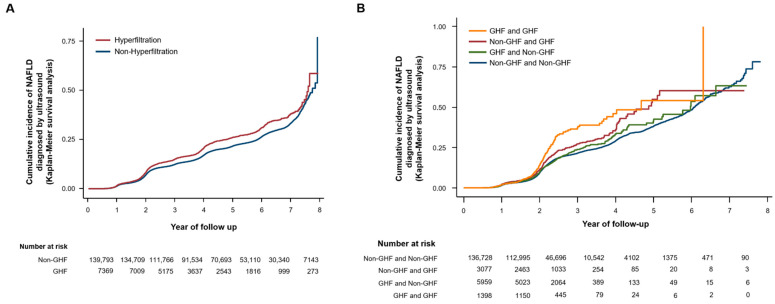
Cumulative incidence of NAFLD by glomerular hyperfiltration status at baseline (**A**) and cumulative incidence of NAFLD by glomerular hyperfiltration status at baseline and at the end of the follow-up (**B**). GHF: glomerular hyperfiltration, NAFLD: nonalcoholic fatty liver disease, Non-GHF: non-glomerular hyperfiltration.

After adjusting for confounders related to obesity and IR, the HR for the development of NAFLD tended to increase as the baseline eGFR percentile increased (Table 2 and Figure 2A). In particular, subjects with hyperfiltration had the highest HR for the development of NAFLD compared to the reference group (HR = 1.21, 95% CI 1.14–1.29, Model 3). The cumulative incidence of NAFLD continued to be higher during the follow-up period in the hyperfiltration group and tended to increase significantly in all percentile groups from the 2nd year of follow-up (Figure 3A). In addition, participants in the higher eGFR percentile at baseline continued to maintain a higher incidence of NAFLD throughout the follow-up period (Supplementary Materials Figure S2). During the follow-up period, 7695 participants had fibrosis progression by NFS criteria. GHF was also significantly associated with the probability of advanced liver fibrosis in NAFLD. The HR for NFS progression tended to increase with increasing baseline eGFR percentiles. Patients with GHF had the highest HR for NFS progression compared to the reference group (HR = 1.42, 95% CI 1.11–1.82, Model 3) (Figure 2B and Table 3). A further sensitivity analysis for the association between GHF and the probability of advanced liver fibrosis by applying the FIB-4 index showed consistent results, although at a lower intensity than NFS (Supplementary Materials Table S4). We performed further analyses with additional adjustments for alcohol intake and ALT (Model 4), but did not find significant differences in the results (Supplementary Materials Tables S2 and S3). 

In the evaluation of the four groups divided by GHF status at baseline and at the end of the follow-up (Table 4), the persistent GHF group had the highest HR for the development of NAFLD compared to the reference group, which was the persistent non-GHF group (HR = 1.31, 95% CI 1.14–1.51, Model 3). This tendency was maintained throughout the follow-up period (Figure 3B).

**Figure 4:**
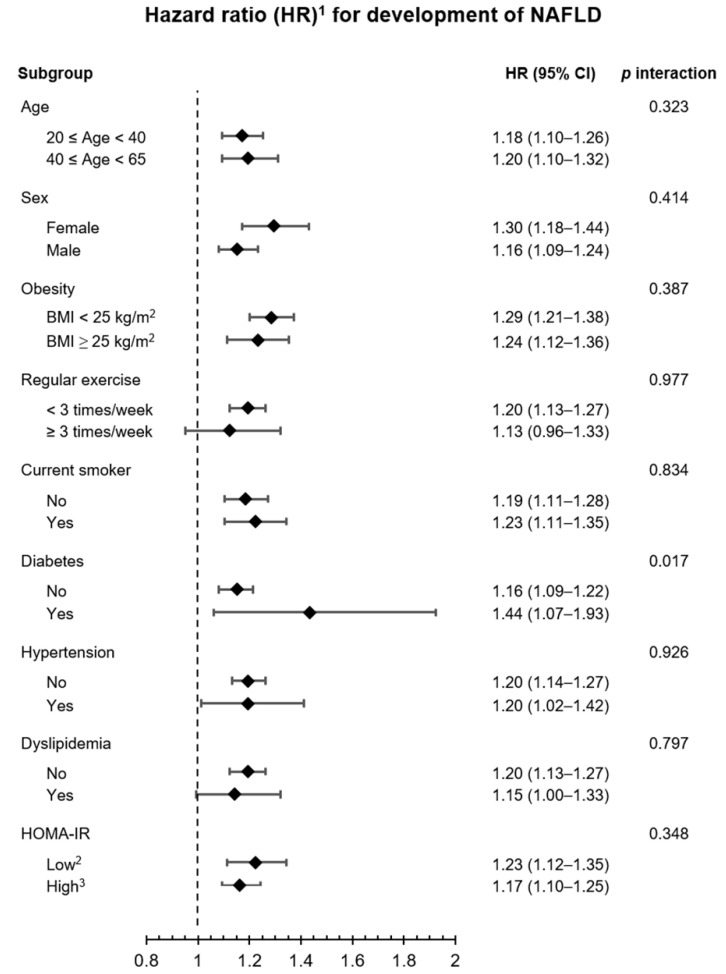
Subgroup analysis for comparing participants with GHF to those without GHF at baseline. ^1^ Adjusted for age, sex, muscle mass, BMI, smoking status, regular exercise, diabetes, dyslipidemia, hypertension, hs-CRP, and HOMA-IR. ^2^ Low HOMA-IR was defined as the value of <50 percentile of study participants. ^3^ High HOMA-IR was defined as the value of ≥50 percentile of study participants. GHF was defined as an eGFR value of ≥95th percentile of study participants in each stratified group. BMI: body mass index, eGFR: estimated glomerular filtration rate, GHF: glomerular hyperfiltration, HOMA-IR: homeostasis model assessment of insulin resistance, hs-CRP: high-sensitivity C-reactive protein, NAFLD: nonalcoholic fatty liver disease.

In subgroup analysis, participants with GHF at baseline maintained the highest HR for the development of NAFLD in all the subgroups (Figure 4). All the *p* values for the interaction were >0.05, except for diabetes. There was an interaction between NAFLD and the presence of diabetes (*p* for interaction = 0.017).

## 4. Discussion

In this longitudinal cohort study, baseline GHF was associated with the development of NAFLD and the probability of advanced liver fibrosis in relatively healthy young individuals, even after adjusting for obesity and IR. We tried to determine the linearity of the relative risk for NAFLD according to the eGFR status, as well as the relationship between renal hyperfiltration status and NAFLD. It was confirmed that the higher the baseline eGFR percentile, the higher the incidence of NAFLD. The hyperfiltration group had the highest incidence of NAFLD, and the 65–79th and 80–94th percentile groups also showed a relatively higher risk of NAFLD than the reference group. In addition, the higher the baseline eGFR percentile, the higher the risk for advanced liver fibrosis. Furthermore, the incidence of NAFLD was influenced not only by the hyperfiltration status at baseline, but also by the hyperfiltration status at the end of the follow-up. Those with persistent hyperfiltration had a higher risk of NAFLD compared to those without persistent hyperfiltration during the follow-up period. This further supports the association between hyperfiltration status and the development of NAFLD based on a large sample and a relatively long follow-up period.

The mechanisms involved in the pathogenesis of GHF have not been fully elucidated. However, GHF and NAFLD share a common pathophysiology and similar phenotype. Thanks to previous studies, it is well known that the degree of liver fibrosis is strongly associated with albuminuria and CKD in patients with NAFLD [32]. Increased IR, which is a major causative factor of NAFLD [33], has also been associated with GHF [13,31,32]. Obesity that induces IR promotes oxidative stress along with chronic inflammation, and dysregulation of adipokines, which are secreted from subcutaneous fat or visceral fat in obese patients and contribute to the development of ectopic fat accumulation in the liver, may be associated with renal hyperfiltration [34]. Obesity also activates the renin–angiotensin and sympathetic nervous systems, which are associated with GHF, IR, and NAFLD [35,36]. Consistent with previous studies, HOMA-IR increased linearly as the eGFR percentile increased in our study, and the hs-CRP levels were higher in the GHF group than in other groups (Supplementary Materials Table S1). This leads to the assumption that IR and inflammation are common denominators of GHF and NAFLD. HT is also associated with the development of NAFLD, and in a retrospective study of U. adults, it was confirmed that the prevalence of NAFLD was higher in patients with HT or high–normal BP than in those with normal BP [37]. However, the positive association between GHF and NAFLD remained independent of obesity, IR and HT in this study. The mechanisms remain unclear, but these results suggest that GHF might be directly linked to NAFLD through other mechanisms that do not fully overlap with those of IR and obesity.

Hepatic function and renal performance share some pathophysiological pathways that are intrinsically linked to each other. The renin–angiotensin system is known to play a key role in this process. Angiotensin II type 1 (AT1) receptor activation in the kidney promotes reactive oxygen species and cytokine production, leading to glomerular efferent arteriole vasoconstriction, glomerulosclerosis and glomerular damage, which can manifest clinically as GHF [11,36]. In the liver, the activation of AT1 receptor leads to the stimulation of new adipogenesis and the inhibition of fatty acid oxidation, which results in the exacerbation of steatosis [38,39]. The activation of the AT1 receptor also induces an increase in IR in the liver and promotes the synthesis of cytokines that induce oxidative stress, inflammation and fibrosis, which may contribute to the whole process of liver damage such as NAFLD and nonalcoholic steatohepatitis (NASH) [38,39]. Based on these physiological mechanisms of NAFLD development, studies on applying an AT1 receptor blocker (ARB) as a therapeutic agent for NAFLD have been conducted and have shown some positive results [40]. For example, telmisartan, candesartan and losartan were shown to be effective therapeutic agents in preventing the exacerbation of NAFLD and NASH [41,42]. In particular, it has been suggested that losartan can prevent the exacerbation of NFLD by inhibiting hypoxia-inducible factor-1α (HIF-1α) in an animal model [43].

The association between GHF and the risk of NAFLD was maintained regardless of sex, age over 40 years, obesity, smoking status, regular exercise performance, DM, HT, dyslipidemia, and the degree of IR. One notable point was that the association between baseline GHF and the development of NAFLD was statistically weak in participants with diabetes. This finding suggests that diabetes and the development of NAFLD are more closely related [3].

This study has several limitations. First, histological validation of NAFLD was not performed. Biopsy is the most reliable method for diagnosing NAFLD. However, biopsy has many practical limitations in a large cohort study of the general population. In particular, problems such as invasiveness, sampling inaccuracy, and cost pose many difficulties in diagnosing NAFLD [44]. In patients with mild steatosis (fat content of less than 12.5%) [45] or in patients with severe obesity (BMI ≥ 35 kg/m^2^) and kidney disease [46,47], ultrasound may have low diagnostic value. However, ultrasound provides relatively accurate information for the diagnosis of NAFLD, especially for the detection of moderate-to-severe fatty liver [48]. In addition, most of the participants in our study had a relatively low BMI (mean BMI = 22.1), and all patients with kidney disease or a history of its related diseases were excluded at baseline. Second, this study was retrospective, and the possibility of selection bias exists. Third, the generalization of our findings to other populations also has some limitations. The data for this study were drawn from a large cohort of Korean workers who were relatively young and healthy adults. Most of them were white-collar workers with a high level of education. Fourth, there was no verification procedure for medication history, especially for use of drugs such as ARBs, and the effects of other drugs and supplements were not completely excluded. Finally, the GFR applied in this study was not measured directly but estimated using creatinine. This implies the possibility of overestimating the actual GFR value.

Despite such limitations, this study has many advantages. The results in this article provide strong evidence for a close relationship between GHF and the development of NAFLD, irrespective of obesity or IR. Because NAFLD is highly affected by obesity, it is essential to distinguish the effects of hyperfiltration alone from those of obesity, IR and HT. Additionally, we aimed to clinically elucidate the association between GHF and fibrosis progression in NAFLD patients. According to the two-hit NAFLD/NASH model [49], the key factors associated with steatohepatitis or fibrosis are underlying oxidative stress and chronic inflammation, which may also be associated with GHF. Furthermore, this study provides a systematic analysis based on large cohort data recorded over a long period of time, which is a significant advantage to improve statistical reliability.

In conclusion, this study demonstrated that GHF was associated with the development of NAFLD and the probability of advanced liver fibrosis in NAFLD diagnosed by USG in relatively young and healthy adults. These results suggest the potential of GHF as a clinical surrogate marker for the early diagnosis of NAFLD regardless of obesity and IR, especially in subjects without diabetes. Further studies using histologically proven or other non-invasive data such as MRI and CT scans will have to be conducted to support these results.

## Figures and Tables

**Table 1:** Baseline characteristics of participants according to the development of NAFLD at the end of the follow-up.

Characteristics	Total	NAFLD		*p*-Value
		No	Yes	
No. of subjects	147,162	117,752	29,410	
Male	70,076 (47.6)	48,528 (41.2)	21,548 (73.3)	<0.001
Age, years	36.3 ± 6.9	36.0 ± 0.0	37.3 ± 0.0	<0.001
BMI, kg/m^2^	22.1 ± 2.7	21.7 ± 0.0	23.9 ± 0.0	<0.001
Obesity	20,792 (14.1)	11,674 (9.9)	9118 (31.0)	<0.001
WC, cm	78.1 ± 8.1	76.7 ± 0.0	83.9 ± 0.1	<0.001
FBG, mg/dL	92.3 ± 10.1	91.7 ± 0.0	95.0 ± 0.1	<0.001
Hemoglobin A1c, %	5.5 ± 0.3	5.5 ± 0.3	5.6 ± 0.4	<0.001
HOMA-IR	1.01 (0.68–1.45)	0.97 (0.65–1.39)	1.2 (0.82–1.71)	<0.001
DM	1649 (1.1)	1039 (0.9)	610 (2.1)	<0.001
SBP, mmHg	106.7 ± 12.2	105.4 ± 0.0	111.7 ± 0.1	<0.001
DBP, mmHg	68.0 ± 9.2	67.2 ± 8.9	71.3 ± 9.4	<0.001
Hypertension	8207 (5.6)	5429 (4.6)	2778 (9.5)	<0.001
AST, U/L	18 (16–22)	18 (15–21)	19 (16–24)	<0.001
ALT, U/L	15 (12–21)	14 (11–20)	19 (14–27)	<0.001
TC, mg/dL	188.1 ± 31.7	186.1 ± 0.1	196.4 ± 0.2	<0.001
Triglyceride, mg/dL	78 (58–108)	73 (56–99)	102 (75–143)	<0.001
LDL, mg/dL	114.2 ± 29.4	111.5 ± 0.1	124.8 ± 0.2	<0.001
HDL, mg/dL	61.8 ± 14.5	63.5 ± 0.0	55.1 ± 0.1	<0.001
Dyslipidemia	15,411 (10.5)	10,804 (9.2)	4607 (15.7)	<0.001
hs-CRP, mg/dL	0.03 (0.02–0.07)	0.03 (0.02–0.06)	0.05 (0.03–0.09)	<0.001
Creatinine, mg/dL	0.8 (0.7–1.0)	0.8 (0.7- 0.9)	0.9 (0.8–1.0)	<0.001
eGFR, mL/min/1.73 m^2^	106.5 (94.0–114.5)	107.9 (95.1–115.0)	100.6 (90.5–111.5)	<0.001
Alcohol intake, g/day	4 (2–11)	4 (1–10)	7 (3–20)	<0.001
Current smoker	24,397 (16.6)	16,029 (13.6)	8368 (28.5)	<0.001
Regular exercise	18,543 (12.6)	14,595 (12.4)	3948 (13.4)	<0.001
NFS	−3.058 ± 0.002	−3.077 ± 0.003	−2.98 ± 0.006	<0.001
FIB-4	0.69 (0.56–0.87)	0.69 (0.56–0.88)	0.68 (0.54–0.85)	<0.001

Values are expressed as *n* (%), mean ± standard deviation, percentage, or median (interquartile range). ALT: alanine aminotransferase, AST: aspartate aminotransferase, BMI: body mass index, DM: diabetes mellitus, eGFR: estimated glomerular filtration rate, FBG: fasting blood glucose, FIB-4: fibrosis-4, HDL: high-density lipoprotein, HOMA-IR: homeostasis model assessment of insulin resistance, hs-CRP: high-sensitivity C-reactive protein, LDL: low-density lipoprotein, NAFLD: nonalcoholic fatty liver disease, NFS: NAFLD fibrosis score, SBP: systolic blood pressure, TC: total cholesterol, WC: waist circumference.

**Table 2:** Incidence and hazard ratios for the development of NAFLD according to eGFR percentile groups.

Percentile Group of eGFR	No. of Participants	Cases, *n*	IR, per 1000 PY	HR (95% CI)		
				Model 1	Model 2	Model 3
<5	7307	1462	46.09 (43.79–48.51)	0.85 (0.80–0.90)	0.88 (0.83–0.93)	0.88 (0.83–0.94)
5–19	22,073	4436	47.02 (45.66–48.43)	0.91 (0.87–0.95)	0.94 (0.90–0.98)	0.94 (0.90–0.98)
20–34	22,088	4478	47.63 (46.26–49.05)	0.95 (0.91–0.99)	0.96 (0.92–1.01)	0.97 (0.93–1.01)
35–49	22,087	4445	48.51 (47.1–49.95)	0.99 (0.95–1.03)	0.99 (0.95–1.03)	0.99 (0.95–1.04)
50–64	22,021	4245	47.34 (45.94–48.78)	1 (reference)	1 (reference)	1 (reference)
65–79	22,164	4514	51.03 (49.56–52.54)	1.09 (1.04–1.13)	1.06 (1.02–1.11)	1.07 (1.02–1.11)
80–94	22,053	4402	52.71 (51.17–54.29)	1.17 (1.12–1.22)	1.11 (1.07–1.16)	1.11 (1.07–1.16)
≥95	7369	1428	56.26 (53.41–59.25)	1.35 (1.27–1.44)	1.21 (1.14–1.28)	1.21 (1.14–1.29)

Model 1: adjusted for age, sex, center, and muscle mass. Model 2: additionally adjusted for BMI, smoking status, regular exercise, history of diabetes, dyslipidemia, and hypertension. Model 3: additionally adjusted for hs-CRP and HOMA-IR. BMI: body mass index, CI: confidence interval, eGFR: estimated glomerular filtration rate, HOMA-IR: homeostasis model assessment of insulin resistance, HR: hazard ratio, hs-CRP: high-sensitivity C-reactive protein, IR: incidence rate, NAFLD: nonalcoholic fatty liver disease, PY: person years.

**Table 3:** Risk of advanced liver fibrosis based on NFS according to the eGFR percentile in patients with NAFLD.

Percentile Group of eGFR	No. of Participants	Cases, *n*	IR, Per 1000 PY	HR (95% CI)		
				Model 1	Model 2	Model 3
<5	512	136	65.21 (55.13–77.15)	0.95 (0.77–1.17)	0.95 (0.77–1.18)	0.96 (0.78–1.19)
5–19	1382	349	61.38 (55.27–68.17)	0.97 (0.82–1.14)	1.03 (0.88–1.21)	1.05 (0.89–1.24)
20–34	1236	301	58.85 (52.57–65.89)	0.95 (0.80–1.12)	1.03 (0.87–1.22)	1.05 (0.88–1.25)
35–49	1185	296	61.31 (54.71–68.71)	1.02 (0.86–1.21)	1.08 (0.91–1.28)	1.09 (0.92–1.29)
50–64	1037	252	58.77 (51.94–66.49)	1 (reference)	1 (reference)	1 (reference)
65–79	1063	259	61.24 (54.22–69.17)	1.03 (0.87–1.23)	1.06 (0.89–1.26)	1.08 (0.90–1.29)
80–94	976	253	66.92 (59.16–75.69)	1.18 (0.99–1.41)	1.11 (0.94–1.33)	1.15 (0.97–1.38)
≥95	304	83	77.36 (62.39–95.93)	1.50 (1.17–1.93)	1.37 (1.07–1.76)	1.42 (1.11–1.82)

Model 1: adjusted for age, sex, center, and muscle mass. Model 2: additionally adjusted for BMI, smoking status, regular exercise, history of diabetes, dyslipidemia, and hypertension. Model 3: additionally adjusted for hs-CRP and HOMA-IR. BMI: body mass index, CI: confidence interval, eGFR: estimated glomerular filtration rate, HOMA-IR: homeostasis model assessment of insulin resistance, HR: hazard ratio, hs-CRP: high-sensitivity C-reactive protein, IR: incidence rate, NAFLD: nonalcoholic fatty liver disease, NFS: NAFLD fibrosis score, PY: person years.

**Table 4:** Incidence and hazard ratios for the development of NAFLD according to GHF status at baseline and at the end of the follow-up.

eGFR Status (Baseline and End of Follow-Up)	No. of Participants	Cases, *n*	IR, Per 1000 PY	HR (95% CI)		
				Model 1	Model 2	Model 3
Non-GHF and Non-GHF ^1^	130,728	12,910	51.74 (50.85–52.64)	1 (reference)	1 (reference)	1 (reference)
Non-GHF and GHF	3077	368	66.25 (59.82–73.38)	1.23 (1.11–1.37)	1.18 (1.07–1.31)	1.18 (1.07–1.31)
GHF and Non-GHF	5959	590	54.21 (50.01–58.76)	1.24 (1.14–1.35)	1.18 (1.08–1.28)	1.17 (1.08–1.28)
GHF and GHF	1398	194	79.08 (68.70–91.02)	1.44 (1.25–1.66)	1.31 (1.14–1.51)	1.31 (1.14–1.51)

^1^ GHF was defined as an eGFR value of ≥95th percentile of the study participants. Model 1: adjusted for age, sex, center, and muscle mass. Model 2: additionally adjusted for BMI, smoking status, regular exercise, history of diabetes, dyslipidemia, and hypertension. Model 3: additionally adjusted for hs-CRP and HOMA-IR. BMI: body mass index, eGFR: estimated glomerular filtration rate, GHF: glomerular hyperfiltration, HOMA-IR: homeostasis model assessment of insulin resistance, HR: hazard ratio, hs-CRP: high-sensitivity C-reactive protein, IR: incidence rate, NAFLD: nonalcoholic fatty liver disease, Non-GHF: non-glomerular hyperfiltration, PY: person years.

## Data Availability

The data are available from the Kangbuk Samsung Health Study, whose authors may be contacted through the corresponding author. Unfortunately, the data are not available to be shared publicly, as we do not have IRB permission for distributing the data. For additional information, please visit the Kangbuk Samsung Health Study at http://kscs.kbsmc.co.kr/ or contact Gayoung Lim (gayoung.lim@samsung.com).

## Supplementary Materials

The following supporting information can be downloaded at: https://www.mdpi.com/article/10.3390/jpm12071142/s1. Figure S1: Distribution of eGFR corresponding to the definition of hyperfiltration according to age in (A) males and (B) females; Figure S2: Cumulative incidence of NAFLD according to eGFR percentile groups at baseline during the follow-up period; Table S1: Baseline characteristics of the participants according to eGFR percentile groups; Table S2: Incidence and hazard ratios for development of NAFLD according to eGFR percentile groups; Table S3: Risk of advanced liver fibrosis based on NFS according to eGFR percentile in patients with NAFLD; Table S4: Risk of advanced liver fibrosis based on FIB-4 by hyperfiltration status in patient with NAFLD.
