# Supplementary material for: Increased Risk of NAFLD in Adults with Glomerular Hyperfiltration: An 8-Year Cohort Study Based on 147,162 Koreans
Source: J Pers Med. 2022 Jul 14;12(7):1142. doi: 10.3390/jpm12071142 (PMC9320347; doi:10.3390/jpm12071142)
Supplement: Supplementary file 1 [file jpm-12-01142-s001.zip › jpm-1806021-supplementary.pdf]

Supplementary Materials

Table S1. Baseline characteristics of participants according to eGFR percentile groups.

Characteristics	eGFR percentile groups								*p* Value
	<5	5-19	20-34	35-49	50-64	65-79	80-95	95≤	
No. of subjects	7307	22,073	22,088	22,087	22,021	22,164	22,053	7369	
Male	3487 (47.7)	10,509 (47.6)	10,539 (47.7)	10,470 (47.4)	10,526 (47.8)	10,533 (47.5)	10,513 (47.7)	3499 (47.5)	<0.001
Age, year	36.8±7.1	36.7±7.0	36.8±6.8	36.4±6.9	35.7±7.0	36.5±6.7	35.6±6.9	35.5±7.0	<0.001
BMI, kg/m^2	22.4±2.8	22.2±2.7	22.2±2.7	22.1±2.7	22.0±2.7	22.0±2.7	22.0±2.7	22.1±2.8	<0.001
Obesity	1275 (17.5)	3374 (15.3)	3200 (14.5)	3133 (14.2)	2959 (13.5)	2910 (13.1)	2882 (13.1)	1059 (14.4)	<0.001
WC, cm	78.3±8.5	78.1±8.2	78.2±7.9	78.0±8.1	78.0±8.1	78.0±7.9	78.0±8.2	78.4±8.3	<0.001
FBG, mg/dL	92.5±10.7	92.4±9.2	92.5±9.5	92.3±9.3	92.3±9.9	92.4±10.4	92.2±11.0	92.2±12.9	0.009
Hemoglobin A1c, %	5.6±0.3	5.5±0.3	5.5±0.3	5.5±0.3	5.5±0.3	5.5±0.3	5.5±0.4	5.5±0.5	<0.001
HOMA-IR	0.97 (0.66-1.40)	0.98 (0.66-1.40)	0.99 (0.67-1.42)	1.01 (0.68-1.44)	1.01 (0.68-1.46)	1.01 (0.68-1.45)	1.05 (0.70-1.49)	1.09 (0.72-1.58)	<0.001
DM	91 (1.3)	192 (0.9)	238 (1.1)	205 (0.9)	226 (1.0)	280 (1.3)	91 (1.3)	192 (0.9)	<0.001
SBP, mmHg	107.6±12.8	107.0±12.4	106.8±12.3	106.8±12.3	106.5±12.1	106.4±12.1	106.3±12.0	106.4±12.5	<0.001
DBP, mmHg	68.91±9.4	68.37±9.2	68.23±9.2	68.07±9.2	67.94±9.1	67.79±9.0	67.62±9.1	68.91±9.4	<0.001
Hypertension	569 (7.8)	1311 (5.9)	1235 (5.6)	1216 (5.5)	1201 (5.5)	1150 (5.2)	569 (7.8)	1311 (5.9)	<0.001
AST, IU/L	19 (16-23)	19 (16-22)	18 (16-22)	18 (16-22)	18 (16- 22)	18 (15-22)	18 (15-21)	18 (15-22)	<0.001
ALT, IU/L	15 (12-21)	15 (12-21)	15 (12-21)	15 (12-21)	15 (11-21)	15 (12-22)	15 (11-21)	16 (12-23)	<0.001
TC, mg/dL	193.9±32.6	191.2±32.1	189.6±31.6	188.7±31.3	187.7±31.2	186.4±31.2	184.8±31.3	183.8±33.3	<0.001
Triglyceride, mg/dL	79 (60-109)	78 (58-107)	78 (58-107)	77 (58-107)	77 (58-106)	78 (58-108)	77 (57-109)	81 (59-116)	<0.001
LDL, mg/dL	117.8±30.6	115.8±29.9	115.3±29.5	114.6±29.2	113.6±29.1	113.3±28.8	112.1±28.9	111.4±29.7	<0.001
HDL, mg/dL	62.4±15.2	62.2±14.6	61.7±14.3	61.7±14.4	62.0±14.5	61.6±14.4	61.6±14.5	62.0±15.0	<0.001
Dyslipidemia	986 (13.5)	2563 (11.6)	2441 (11.1)	2269 (10.3)	2217 (10.1)	2148 (9.7)	986 (13.5)	2563 (11.6)	<0.001
hs-CRP, mg/dL	0.03 (0.02-0.06)	0.03 (0.02-0.06)	0.03 (0.02-0.06)	0.03 (0.02-0.06)	0.03 (0.02-0.07)	0.03 (0.02-0.07)	0.04 (0.02-0.07)	0.04 (0.02-0.08)	<0.001
Creatinine, mg/dL	1.0 (0.9-1.2)	0.8 (0.8-1.1)	0.8 (0.7-1.0)	0.7 (0.7-1.0)	0.7 (0.7-1.0)	0.7 (0.6-0.9)	0.6 (0.6-0.8)	0.6 (0.5-0.8)	<0.001
eGFR, mL/min/1.73m^2	78.6±7.0	89.2±7.2	98.5±10.5	103.8±9.00	107.4±8.4	112.3±6.6	115.4±6.6	121.4±7.7	<0.001
Alcohol intake, g/day	4 (2-10)	4 (2-11)	4 (2-11)	4 (2-11)	4 (2-11)	4 (2-11)	4 (2-13)	4 (2-13)	0.040
Current smoker	1035 (14.2)	3398 (15.4)	3620 (16.4)	3546 (16.1)	3706 (16.8)	3941 (17.8)	3867 (17.5)	1284 (17.4)	<0.001
Exercise status									<0.001
< 3 times/week	5811 (79.5)	18,150 (82.2)	18,454 (83.6)	18,728 (84.8)	18,791 (85.3)	18,963 (85.6)	19,122 (86.7)	6438 (87.4)	
≥ 3 times/week	1249 (17.1)	3228 (14.6)	2976 (13.5)	2688 (12.2)	2666 (12.1)	2602 (11.7)	2377 (10.8)	757 (10.3)	

NFS	-2.90±0.96	-2.96±0.94	-3.00±0.94	-3.04±0.95	-3.09±0.94	-3.09±0.94	-3.17±0.96	-3.20±0.97	<0.001
FIB-4	0.75 (0.6-0.94)	0.72 (0.58-0.91)	0.71 (0.57-0.89)	0.7 (0.56-0.87)	0.68 (0.55-0.85)	0.68 (0.55-0.86)	0.65 (0.53-0.82)	0.64 (0.51-0.81)	<0.001

Values are expressed as n (%), mean±standard deviation, percentage, or median (interquartile range).

ALT: alanine aminotransferase, AST: aspartate aminotransferase, BMI: body mass index, DM: diabetes mellitus, eGFR: estimated glomerular filtration rate, FBG: fasting blood glucose, FIB-4: fibrosis-4, HDL: high density lipoprotein, hs-CRP: high-sensitivity C-reactive protein, HOMA-IR: homeostasis model assessment of insulin resistance, LDL: low density lipoprotein, NAFLD: nonalcoholic fatty liver disease, NFS: NAFLD fibrosis score, SBP: systolic blood pressure, TC: total cholesterol, WC: waist circumference.

Table S2. Incidence and hazard ratios for development of NAFLD according to eGFR percentile groups.

Percentile Group of eGFR	No. of Participants	Cases, *n*	IR, per 1000 PY	HR (95% CI)			
				Model 1	Model 2	Model 3	Model 4
**<5**	**7307**	**1462**	46.09 (43.79–48.51)	0.85 (0.80–0.90)	0.88 (0.83–0.93)	0.88 (0.83–0.94)	0.89 (0.84-0.95)
5–19	22,073	4436	47.02 (45.66–48.43)	0.91 (0.87–0.95)	0.94 (0.90–0.98)	0.94 (0.90–0.98)	0.95 (0.91-0.99)
20–34	22,088	4478	47.63 (46.26–49.05)	0.95 (0.91–0.99)	0.96 (0.92–1.01)	0.97 (0.93–1.01)	0.98 (0.93-1.02)
35–49	22,087	4445	48.51 (47.1–49.95)	0.99 (0.95–1.03)	0.99 (0.95–1.03)	0.99 (0.95–1.04)	0.99 (0.95-1.03)
50–64	22,021	4245	47.34 (45.94–48.78)	1 (reference)	1 (reference)	1 (reference)	1 (reference)
65–79	22,164	4514	51.03 (49.56–52.54)	1.09 (1.04–1.13)	1.06 (1.02–1.11)	1.07 (1.02–1.11)	1.07 (1.03-1.12)
80–94	22,053	4402	52.71 (51.17–54.29)	1.17 (1.12–1.22)	1.11 (1.07–1.16)	1.11 (1.07–1.16)	1.12 (1.07-1.17)
≥95	7369	1428	56.26 (53.41–59.25)	1.35 (1.27–1.44)	1.21 (1.14–1.28)	1.21 (1.14–1.29)	1.20(1.13-1.28)

Model 1: adjusted for age, sex, center, and muscle mass. Model 2: additionally adjusted for BMI, smoking status, regular exercise, history of diabetes, dyslipidemia, and hypertension. Model 3: additionally adjusted for hs-CRP and HOMA-IR. Model 4: additionally adjusted for alcohol intake and ALT. BMI: body mass index, CI: confidence interval, eGFR: estimated glomerular filtration rate, HOMA-IR: homeostasis model assessment of insulin resistance, HR: hazard ratio, hs-CRP: high-sensitivity C-reactive protein, IR: incidence rate, NAFLD: nonalcoholic fatty liver disease, PY: person years.

Table S3. Risk of advanced liver fibrosis based on NFS according to eGFR percentile in patients with NAFLD.

Percentile Group of eGFR	No. of Participants	Cases, *n*	IR, per 1000 PY	HR (95% CI)			
				Model 1	Model 2	Model 3	Model 4
**<5**	**512**	**136**	65.21 (55.13–77.15)	0.95 (0.77–1.17)	0.95 (0.77–1.18)	0.96 (0.78–1.19)	1.01 (0.81-1.26)
5–19	1382	349	61.38 (55.27–68.17)	0.97 (0.82–1.14)	1.03 (0.88–1.21)	1.05 (0.89–1.24)	1.06 (0.89-1.27)
20–34	1236	301	58.85 (52.57–65.89)	0.95 (0.80–1.12)	1.03 (0.87–1.22)	1.05 (0.88–1.25)	1.06 (0.88-1.26)
35–49	1185	296	61.31 (54.71–68.71)	1.02 (0.86–1.21)	1.08 (0.91–1.28)	1.09 (0.92–1.29)	1.10 (0.92-1.32)
50–64	1037	252	58.77 (51.94–66.49)	1 (reference)	1 (reference)	1 (reference)	1 (reference)
65–79	1063	259	61.24 (54.22–69.17)	1.03 (0.87–1.23)	1.06 (0.89–1.26)	1.08 (0.90–1.29)	1.12 (0.93-1.34)
80–94	976	253	66.92 (59.16–75.69)	1.18 (0.99–1.41)	1.11 (0.94–1.33)	1.15 (0.97–1.38)	1.17 (0.97-1.41)
≥95	304	83	77.36 (62.39–95.93)	1.50 (1.17–1.93)	1.37 (1.07–1.76)	1.42 (1.11–1.82)	1.43 (1.10-1.86)

Model 1: adjusted for age, sex, center, and muscle mass. Model 2: additionally adjusted for BMI, smoking status, regular exercise, history of diabetes, dyslipidemia, and hypertension. Model 3: additionally adjusted for hs-CRP and HOMA-IR. Model 4: additionally adjusted for alcohol intake and ALT. BMI: body mass index, CI: confidence interval, eGFR: estimated glomerular filtration rate, HOMA-IR: homeostasis model assessment of insulin resistance, HR: hazard ratio, hs-CRP: high-sensitivity C-reactive protein, IR: incidence rate, NAFLD: nonalcoholic fatty liver disease, PY: person years.

Table S4. Risk of advanced liver fibrosis based on FIB-4 by hyperfiltration status in patient with NAFLD.

eGFR status at baseline	No. of participants	Cases, n	IR, per 1,000 PY	HR (95% CI)		
				Model 1	Model 2	Model 3
Non-GHF[a]	6,446	1,128	44.02 (41.52-46.66)	1 (reference)	1 (reference)	1 (reference)
GHF[a]	304	83	77.36 (62.39-95.93)	1.14 (0.85-1.54)	1.17 (0.87-1.57)	1.21 (0.90-1.64)

[a] GHF was defined as an eGFR value above the 95th percentile for each sex and age group of study participants.

Model 1: adjusted for age, sex, center, and muscle mass.

Model 2: additionally adjusted for BMI, smoking status, regular exercise, history of diabetes, dyslipidemia, and hypertension.

Model 3: additionally adjusted for hs-CRP and HOMA-IR.

BMI, body mass index; CI, confidence interval; eGFR, estimated glomerular filtration rate; FIB-4, fibrosis-4; GHF, glomerular hyperfiltration; HOMA-IR, homeostasis model assessment of insulin resistance; HR, hazard ratio; hs-CRP, high-sensitivity C-reactive protein; IR, incidence rate; NAFLD, nonalcoholic fatty liver disease; NFS, NAFLD fibrosis score; Non-GHF, non- glomerular hyperfiltration; PY person-years.

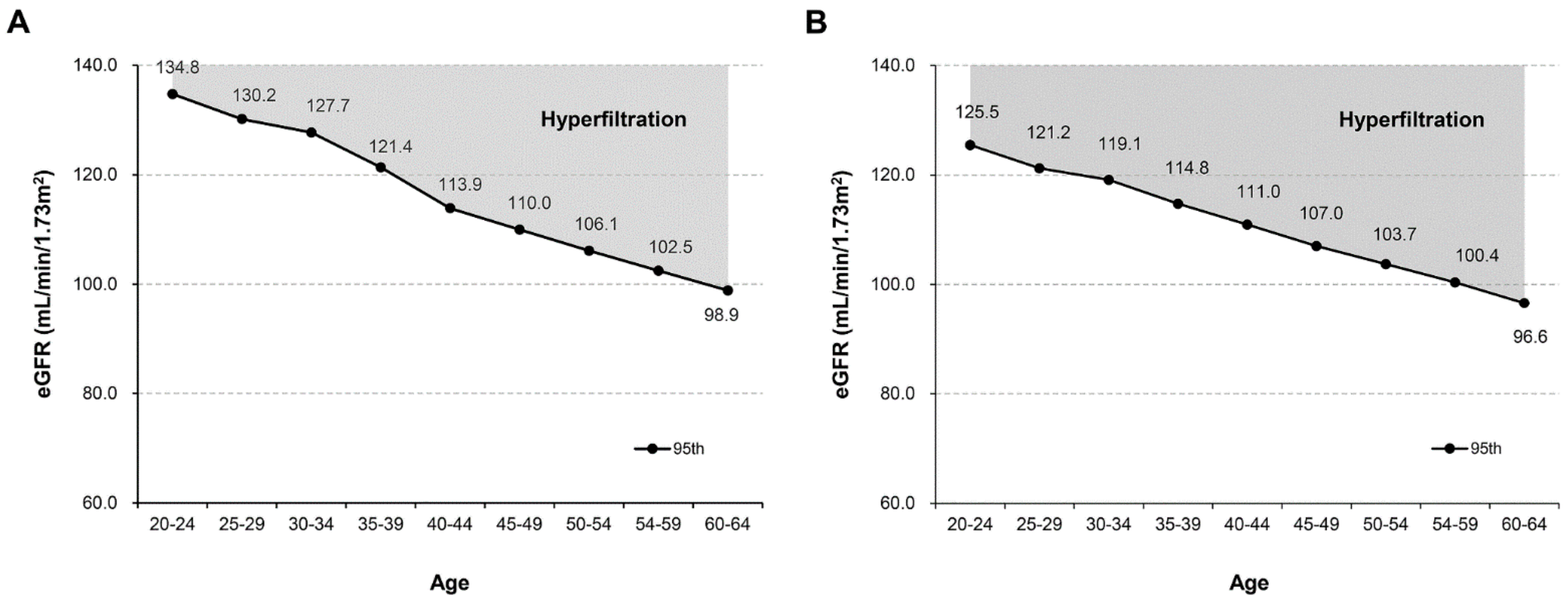

Figure S1. Distribution of eGFR corresponding to the definition of hyperfiltration according to age in (A) male and (B) female.

eGFR: estimated glomerular filtration rate.

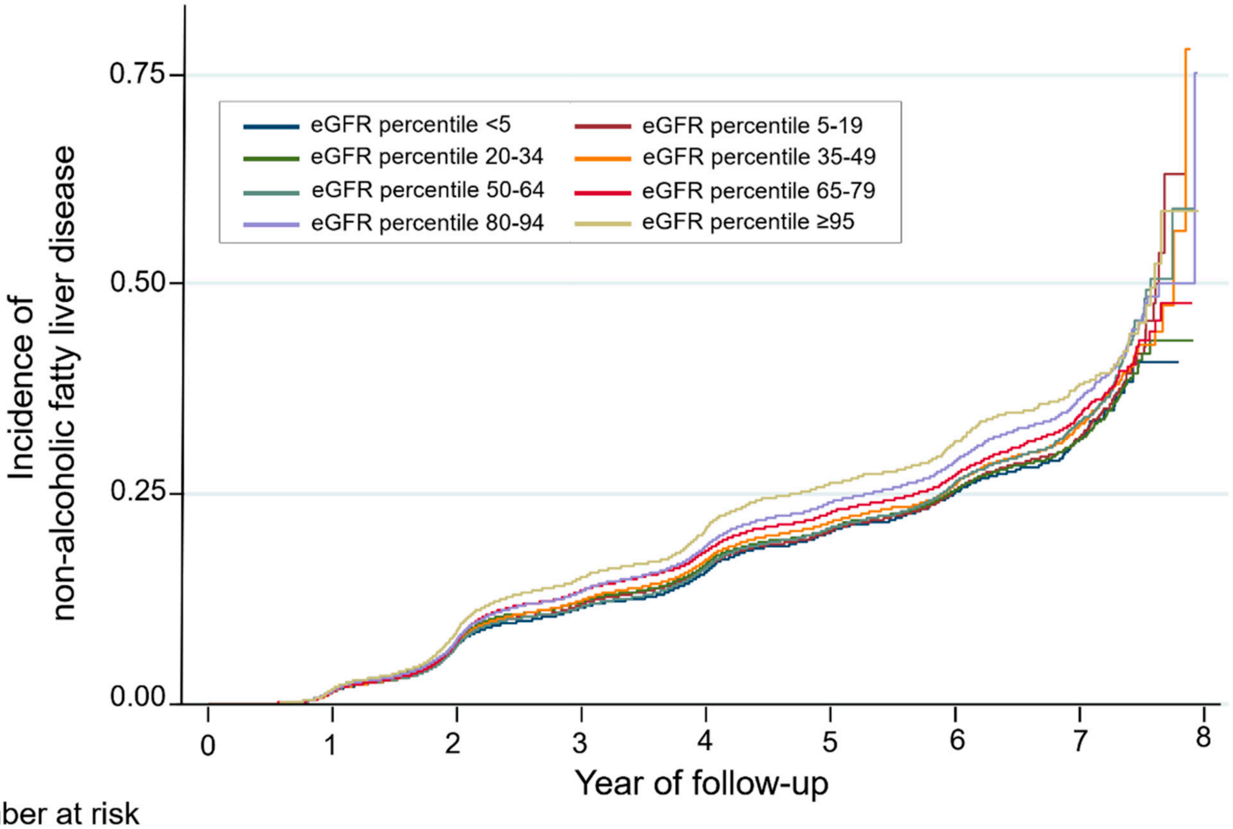

Number at risk

eGFR percentile <5	7,333	7,069	6,095	5,221	4,155	3,174	1,810	394
eGFR percentile 5-19	22,103	21,332	18,166	15,445	12,137	9,255	5,205	1,128
eGFR percentile 20-34	22,213	21,416	18,083	15,205	12,030	9,236	5,534	1,402
eGFR percentile 35-49	22,047	21,277	17,851	14,697	11,397	8,622	4,822	1,037
eGFR percentile 50-64	22,156	21,409	17,769	14,550	11,083	8,129	4,474	1,064
eGFR percentile 65-79	22,130	21,293	17,363	13,691	10,555	7,939	4,676	1,151
eGFR percentile 80-94	22,109	21,207	16,663	12,880	9,439	6,831	3,861	978
eGFR percentile ≥95	7,388	7,027	5,168	3,623	2,511	1,774	980	268

Figure S2. Cumulative incidence of NAFLD according to eGFR percentile groups at baseline during the follow-up period. NAFLD: nonalcoholic fatty liver disease, eGFR: estimated glomerular filtration rate.
